# Novel Polyamides with 5*H*-Dibenzo[*b*,*f*]azepin-5-yl-Substituted Triphenylamine: Synthesis and Visible-NIR Electrochromic Properties

**DOI:** 10.3390/polym9100542

**Published:** 2017-10-23

**Authors:** Qingyi Lu, Wanan Cai, Haijun Niu, Wen Wang, Xuduo Bai, Yanjun Hou

**Affiliations:** 1 Key Laboratory of Functional Inorganic Material Chemistry, Ministry of Education, Department of Macromolecular Science and Engineering, School of Chemical, Chemical Engineering and Materials, Heilongjiang University, Harbin 150086, China; qingyiluhlju@163.com (Q.L.); w.a.cai@hrbeu.edu.cn (W.C.); baixuduo@hlju.edu.cn (X.B.); houyj@hlju.edu.cn (Y.H.); 2 School of Materials Science and Engineering, Harbin Institute of Technology, Harbin 150080, China

**Keywords:** triphenylamine, 5*H*-dibenzo[*b,f*]azepine, polyamide, electrochemical

## Abstract

In this study, a new diamine monomer, namely 4,4′-diamino-4″-(5*H*-dibenzo[*b,f*]azepin-5-yl)triphenylamine, was prepared and polymerized with four kinds of dicarboxylic acids via direct polycondensation reaction resulting in a novel series of soluble and electroactive polyamides (PAs). The tough thin films of all PAs could be solution-cast onto an indium-tin oxide (ITO)-coated glass substrate owing to the good solubility in polar organic solvents. Two pairs of obvious redox peaks for these films were observed in cyclic voltammetry (CV) with low onset potentials (*E*_onset_) of 0.37–0.42 V accompanying with remarkable reversible color changes between light yellow and dark blue. A new absorption peak at around 915 nm emerged in near infrared (NIR) spectra; the increasing potential indicated that PAs could be used as a NIR electrochromic material. Moreover, the PAs showed high coloration efficiency (CE; η) in the range of 190–259 cm^2^ C^−1^.

## 1. Introduction

Due to their significant, lasting, and reversible changes in color upon reduction or oxidation, electrochromic materials have received a great deal of attention and also play an important role in our lives via electrochromic windows, e-paper, electrochromic energy storage devices, and adaptive camouflage [1,2,3,4]. There are many chemical species frequently used for electrochromic studies, including metal coordination complexes, metal oxides, and conducting polymers (such as polyanilines) [5,6]. However, conjugated polymers always have a deep color in neutral state, which limits their use in smart window that will block the light in a dark environment. In recent years, triarylamine-based condensation-type polymers such as aromatic PAs and polyimides (PIs) have been developed to resolve the problem, and have been reported as a new and attractive family of electrochromic materials due to their mechanical flexibility, high optical contrast ratios, and long-term redox stability [7,8,9,10].

It is common knowledge that aromatic PAs have many outstanding material properties, such as high thermal stability, good electrical properties, excellent mechanical properties, and chemical resistance [11,12,13]. However, due to their rigid backbones and strong interchain interactions, most aromatic PAs have a high melting or softening temperature. To overcome these drawbacks, Wang et al. offer a strategy to incorporate flexible linkages, asymmetric units, or bulky pendant groups into the Pas’ backbones [14,15,16]. In the past few years, Liou and Hsiao’s groups have reported a large number of triarylamine-based high-performance polymers [17,18,19,20,21,22]. The introduction of propeller-shaped triarylamine units into the PAs backbone as a structural modification to rigid PIs has the potential to form an amorphous structure exhibiting excellent solubility and film-forming capabilities [23,24,25].

On the other hand, triphenylamine (TPA) and its derivatives are widely used in hole transport materials in organic photo-electronic devices because of their stable radical cations and good hole mobility [26,27,28]. The introduction of TPA units into the polymers could enhance the glass transition temperature (*T*_g_) and solution processability, and also facilitate the charge transfer (CT) behavior of polymers [29,30,31]. Incorporation of electron-donating substituent at the para position of TPA can afford a stable radical cation and enhance the electrochemical and electrochromic stability of the polymers, and the oxidation process is always associated with a noticeable change of color [32,33]. Recently, Feng and Zhu’s groups reported that 5*H*-dibenzo[*b,f*]azepine is used as a dye-sensitized solar cells (DSSCs) [34,35]. 5*H*-dibenzo[*b,f*]azepine is a conjugated structure that will facilitate electron mobility. Inspired by these studies, 5*H*-dibenzo[*b,f*]azepine is expected to promote the electrochemical and electrochromic stability of the resulting PAs. In our study, it was demonstrated that the introduction of 5*H*-dibenzo[*b*,*f*]azepine as an electron donor into the backbone of PAs greatly enhanced the performance of PAs. In particular, the driving voltage of the polymers is significantly reduced, which will greatly promote the development of photoelectric materials.

In this article, a new diamine monomer and a novel series of electroactive aromatic PAs were synthesized. We make a detailed investigation of the properties of these PAs, such as organic solubility, thermal properties, and photoelectric performances.

## 2. Experimental

### 2.1. Materials

4,4′-Sulfonyldibenzoic acid (TCI), 5*H*-dibenzo[*b*,*f*]azepine (TCI), 2,2-bis-(4-carboxyphenyl)-hexafluoropropane (TCI), 1,4-cyclohexanedicarboxylic acid (Sinopharm Chemical Reagent Co., Ltd., Shanghai, China), 1,4-dicarboxybenzene (Sinopharm Chemical Reagent Co., Ltd., Shanghai, China), 4-fluoronitrobenzene (Sinopharm Chemical Reagent Co., Ltd. Shanghai, China), 10% palladium on charcoal (Pd/C, Acros, Geel, Belgium), and 80% hydrazine monohydrate (TCI) were used as received. *N*,*N*-dimethylformamide (DMF) and *N*-methyl-2-pyrrolidinone (NMP) were dried over calcium hydride overnight (for 18 h), distilled under reduced pressure (−0.1 MPa), then stored in molecular sieves in a sealed bottle before use. Tetrabutylammonium perchlorate (Bu_4_NClO_4_, Acros, Geel, Belgium) was recrystallized twice from ethanol under nitrogen atmosphere and then dried before use. Other commercially available chemicals and solvents were used without further purification.

### 2.2. Measurements

Fourier transform infrared (FTIR) spectra were recorded on a PerkinElmer (Fremont, CA, USA) Spectrum 100 Model FT-IR spectrometer. ^1^H NMR (Nuclear Magnetic Resonance), ^13^C NMR, H-H COSY, and C-H HSQC (Heteronuclear Singular Quantum Correlation) spectra were measured by a Bruker (Rheinstetten, Germany) AVANCE 500 FT-NMR system with tetramethylsilane as an internal reference. Gel permeation chromatography (GPC) analysis was performed on a Malvern instrument (Worcestershire, UK) connected with double refractive index detector. Thermogravimetric analysis (TGA) was conducted on a PerkinElmer (Pyris 6 TGA, Fremont, CA, USA) in a nitrogen atmosphere with a heating rate of 10 K × min^−1^ and a sample weight of 8–10 mg. UV-Vis absorption spectra were recorded using a Shimadzu (Kyoto, Japan) UV-3600 spectrophotometer.

CV measurements were carried out on a CH Instruments, Inc (Shanghai, China) 660E electrochemical work station at a scan rate of 50 mV s^−1^. A 0.1 M solution of Bu_4_NClO_4_ in dry acetonitrile (ACN) worked as the supporting electrolyte and a Pt wire and Ag/AgCl electrodes worked as the counter electrode and the reference electrode, respectively. The PA films that were cast on an ITO-coated glass slide were measured. The HOMO (Highest Occupied Molecular Orbital) and LUMO (Lowest Unoccupied Molecular Orbital) levels of PAs were calculated on the premise of the absolute energy level of Fc/Fc^+^ as −4.80 eV. The density functional theory (DFT) is calculated on a computer. Geometric optimization was performed using the B3LYP functional in the Gaussian 03 program.

### 2.3. Synthesis of Monomers

#### 2.3.1. 5-(4-Nitrophenyl)-5*H*-dibenzo[*b*,*f*]azepine (M1)

In a 250-mL three-neck round-bottom flask equipped with a magnetic rotor, a mixture of 2.90 g (15.0 mmol) of 5*H*-dibenzo[*b*,*f*]azepine, 0.53 g (22.0 mmol) of sodium hydride in 110 mL of dried DMF was stirred under a nitrogen atmosphere at 25 °C for 0.5 h. Then, 2.33 g (16.5 mmol) of 4-fluoronitrobenzene was added dropwise at 25 °C. The mixture was stirred in a nitrogen atmosphere at 115 °C for 24 h. After cooling to room temperature, the reaction solution was poured into 500 mL of water to precipitate the crude solid. Recrystallization from ethanol yielded the compound M1 as a yellow powder in 85.5% yield, m.p.: 169–170 °C. FTIR (KBr): 1306, 1586 (–NO_2_ stretch) cm^−1^. ^1^H NMR (400 MHz, DMSO-*d*_6_, δ, ppm): 7.98–7.96 (d, 2H, H_a_), 7.67–7.65 (d, 6H, H_d_ + H_f_ + H_g_), 7.56–7.52 (t, 2H, H_b_), 7.03 (s, 2H, H_e_), 6.26–6.28 (d, 2H, H_c_).

#### 2.3.2. 4-(5*H*-Dibenzo[*b*,*f*]azepin-5-yl)aniline (M2)

First 0.50 g of Pd/C and 3.14 g (10.0 mmol) of M1 were added into 100 mL of ethanol under nitrogen atmosphere in a round-bottom flask of 250 mL capacity. Then, 12.0 mL of hydrazine monohydrate were added dropwise, and the mixture was refluxed at 78 °C for 8 h. Then, the reaction mixture was filtered to remove Pd/C and poured into 300 mL of water; finally, compound M2 was obtained as a white solid in 62.7% yield, m.p.: 137–138 °C. FTIR (KBr): 3203, 3324 (–NH_2_ stretch) cm^−1^. ^1^H NMR (400 MHz, DMSO-*d*_6_, δ, ppm): 7.56–7.50 (m, 4H, H_d_ + H_f_), 7.46–7.44 (t, 2H, H_g_), 7.41–7.37 (t, 2H, H_e_), 6.88 (s, 2H, H_c_), 6.30–6.26 (d, 2H, H_b_), 5.93–5.90 (d, 2H, H_a_), 5.80 (s, 2H, –NH_2_).

#### 2.3.3. 4,4′-Dinitro-4″-(5*H*-dibenzo[*b*,*f*]azepin-5-yl)triphenylamine (M3)

A mixture of 2.84 g (10.0 mmol) of M2, 2.96 g (21.0 mmol) of 4-fluoronitrobenzene, 0.72 g (30.0 mmol) of sodium hydride, and 100 mL of dried DMF were heated under stirring at 115 °C for 24 h. Then, the reaction solution was poured into ice water to precipitate a brown product. Yield 79.2%, m.p.: 138–140 °C. FTIR (KBr): 1306, 1588 (–NO_2_ stretch) cm^−1^. ^1^H NMR (400 MHz, DMSO-*d*_6_, δ, ppm): 8.36–8.34 (d, 6H, H_a_ + H_h_), 8.08–8.06 (d, 4H, H_f_ + H_i_), 7.47–7.37 (d, 6H, H_b_ + H_g_), 6.80–6.78 (d, 6H, H_e_ + H_c_ + H_d_).

#### 2.3.4. 4,4′-Diamino-4″-(5*H*-dibenzo[*b*,*f*]azepin-5-yl)triphenylamine (M4)

In a 250-mL three-neck round-bottomed flask, 1.00 g of Pd/C and 2.63 g (5.00 mmol) of M3 were added into 100 mL of ethanol under a nitrogen atmosphere. Then, 15.0 mL of hydrazine monohydrate were added dropwise, and the mixture was refluxed at 78 °C for 24 h. The solution was filtered to remove Pd/C and poured into 300 mL of water to give a white solid with a yield of 70.7%, m.p.: 160–162 °C. FTIR (KBr): 3210, 3349 (–NH_2_ stretch) cm^−1^. ^1^H NMR (400 MHz, DMSO-*d*_6_, δ, ppm): 7.55–7.50 (m, 6H, H_h_ + H_f_), 7.47–7.45 (d, 4H, H_i_), 7.40–7.36 (t, 2H, H_g_), 6.88 (s, 2H, H_e_) 6.32–6.29 (d, 8H, H_a_ + H_b_) 5.97–5.94 (d, 4H, H_c_ + H_d_) 4.39 (–NH_2_). ^13^CNMR (100 MHz, DMSO-*d*_6_, δ, ppm): 144.43 (C^1^), 140.94 (C^5^ + C^8^), 140.42 (C^9^), 136.89 (C^4^), 131.09 (C^3^ + C^6^ + C^7^), 130.89 (C^15^), 130.86 (C^14^), 130.24 (C^13^), 127.27 (C^12^), 115.03 (C^10^), 113.45 (C^2^).

### 2.4. Synthesis of PAs

The synthesis of PA–a was used as an example to illustrate the general synthetic procedure. In a typical procedure, a mixture of 0.12 g (1.1 mmol) of the 1,4-cyclohexanedicarboxylic acid, 0.51 g (1.1 mmol) of M4, 0.13 g of calcium chloride (CaCl_2_), 1.00 mL of triphenyl phosphite, 0.50 mL of pyridine, and 1.50 mL of NMP was heated with stirring at 120 °C for 3 h. After cooling to room temperature, the solution was poured slowly into 250 mL of methanol, which produced a stringy, fiber-like precipitate. The resulting polymer was washed thoroughly with hot water and methanol, and then with NMP/methanol for further purification (70.8% yield). FTIR (KBr): 3315 (amide N–H stretch), 1667 (amide C=O stretch) cm^−1^. ^1^HNMR (400 MHz, DMSO-*d*_6_, δ, ppm): 9.70 (amide N–H), 8.21-6.65 (aromatic ring of benzene).

Synthesis of PA–b. Yield: 69.6%. FTIR (KBr): 3301 (amide N–H stretch), 1664 (amide C=O stretch) cm^−1^. ^1^HNMR (400 MHz, DMSO-*d*_6_, δ, ppm): 10.26 (amide N–H), 8.18–6.42 (aromatic ring of benzene).

Synthesis of PA–c. Yield: 71.3%. FTIR (KBr): 3313 (amide N–H stretch), 1668 (amide C=O stretch) cm^−1^. ^1^HNMR (400 MHz, DMSO-*d*_6_, δ, ppm): 10.29 (amide N–H), 8.19–6.41 (aromatic ring of benzene).

Synthesis of PA–d. Yield: 78.2%. FTIR (KBr): 3315 (amide N–H stretch), 1665 (amide C=O stretch) cm^−1^. ^1^HNMR (400 MHz, DMSO-*d*_6_, δ, ppm): 10.34 (amide N–H), 8.18–6.71 (aromatic ring of benzene).

## 3. Results and Discussion

### 3.1. Monomer and Polyamide Synthesis

**Scheme 1:**
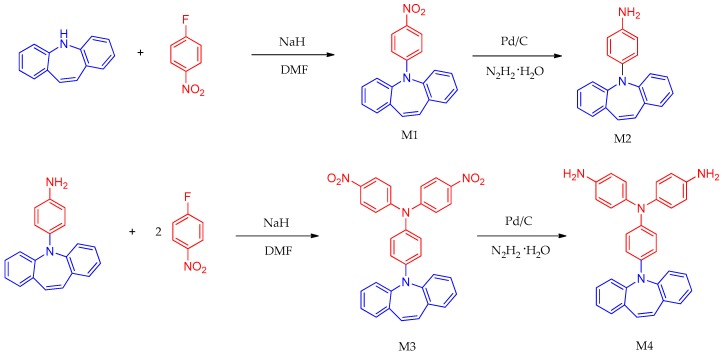
Synthetic route to the diamine monomer M4.

**Figure 1:**
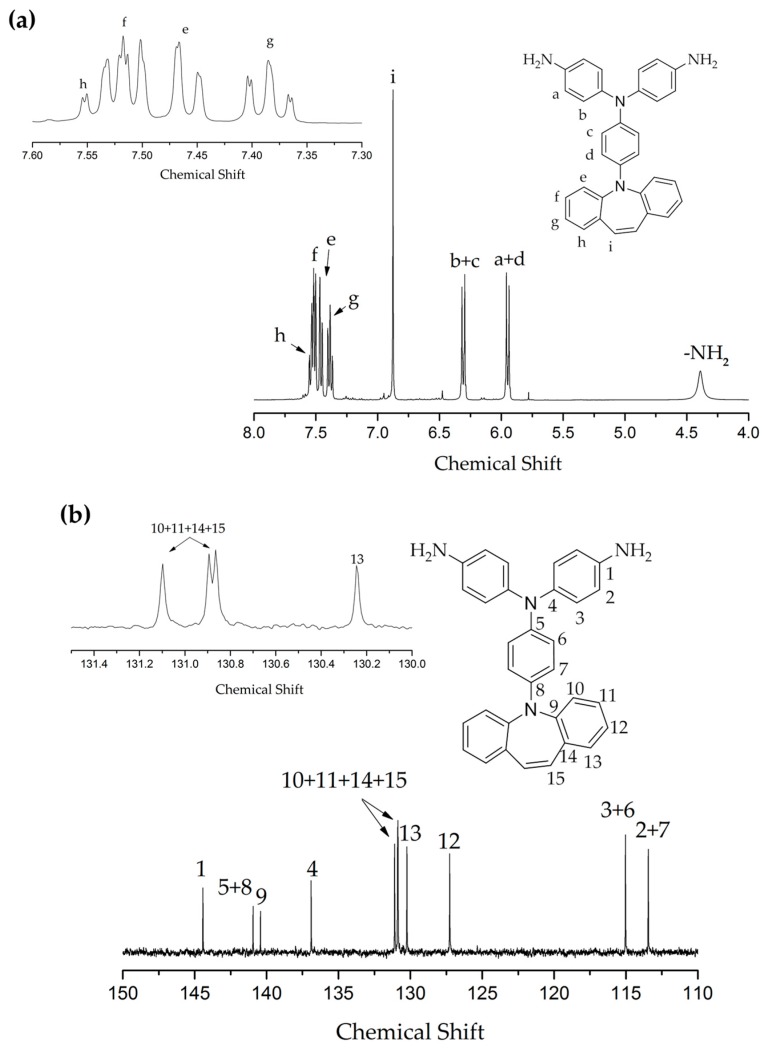

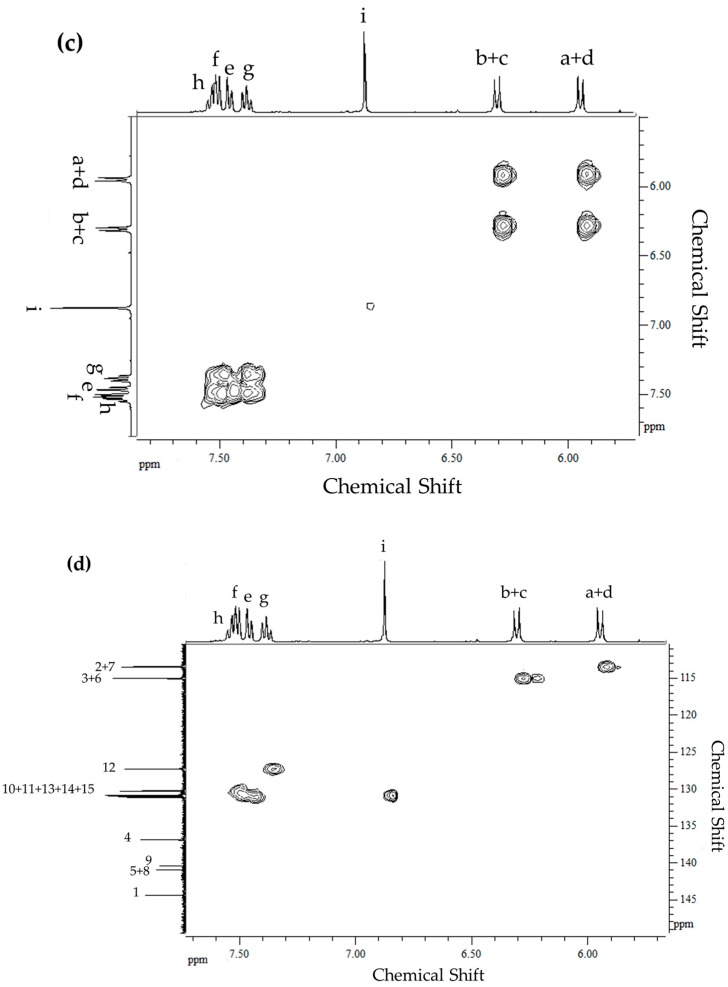
(**a**) ^1^H–NMR; (**b**) ^13^C–NMR; (**c**) H–H COSY; and (**d**) C–H HSQC spectrum of M4 in DMSO-*d*_6_.

**Figure A1:**
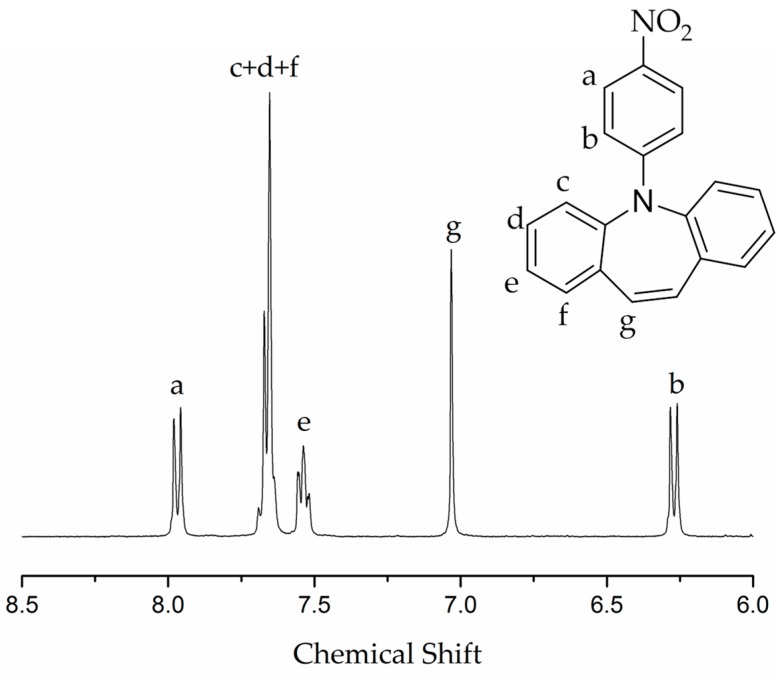
^1^H NMR spectrum of M1 in DMSO-*d*_6_.

**Figure A2:**
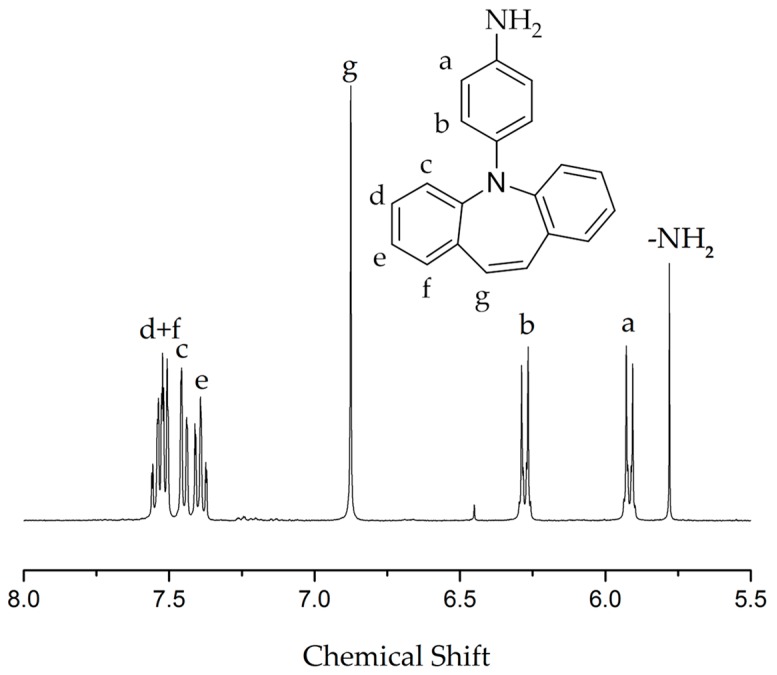
^1^H NMR spectrum of M2 in DMSO-*d*_6_.

**Figure A3:**
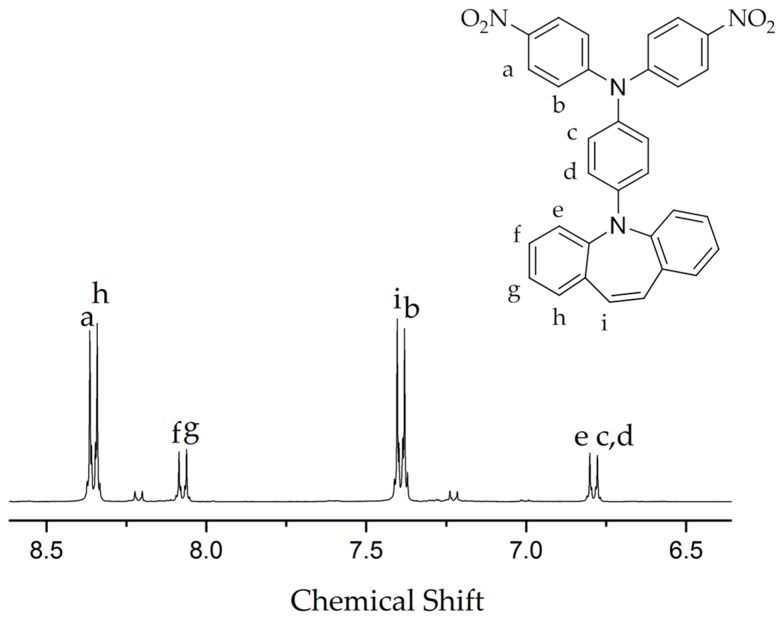
^1^H NMR spectrum of M3 in DMSO-*d*_6_.

The synthetic routes for the monomers are shown in Scheme 1. The new diamine M4 was synthesized via a four-step route. M4 was prepared through the sodium hydride-mediated nucleophilic displacement reaction of M3 with 4-fluoronitrobenzene, followed by a hydrazine Pd/C-catalyzed reduction. FTIR (Figure A8) and ^1^H NMR (Figure A1, Figure A2 and Figure A3) spectroscopic techniques were used to identify the structures of the intermediate compounds M1, M2 and M3. The spectra of IR and ^1^HNMR agree well with the carbon and proton of the proposed molecular structure. To our knowledge, M4 was first reported in this paper, and it was characterized thoroughly. As shown in Figure A8, the characteristic bands of nitro groups at around 1306 and 1588 cm^−1^ disappeared after the reduction reaction. Meanwhile, typical N–H stretching absorptions pairs corresponding to the amino group appeared in the region of 3210 and 3349 cm^−1^. Furthermore, as shown in Figure 1, the ^1^H NMR, ^13^C NMR, H–H COSY, and C–H HSQC spectra of the diamine monomer M4 further confirm that the target diamine monomer has been synthesized successfully.

**Scheme 2:**
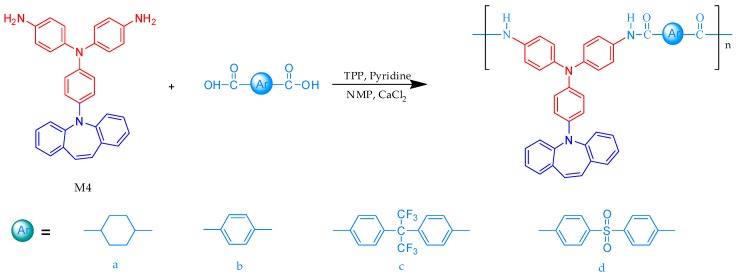
Synthesis of the PAs.

**Figure A4:**
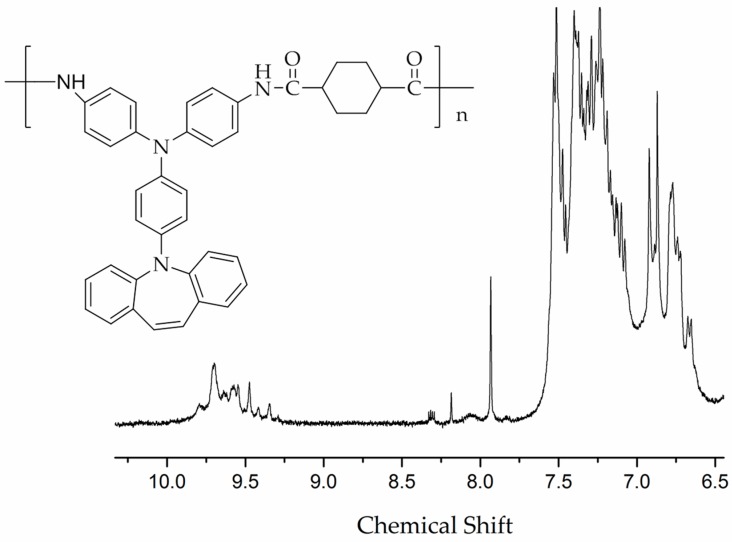
^1^H NMR spectrum of PA–a in DMSO-*d*_6_.

**Figure A5:**
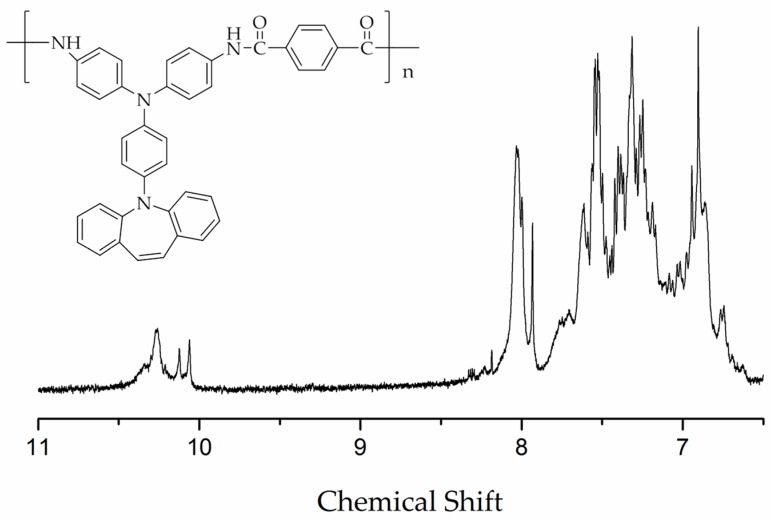
^1^H NMR spectrum of PA–b in DMSO-*d*_6_.

**Figure A6:**
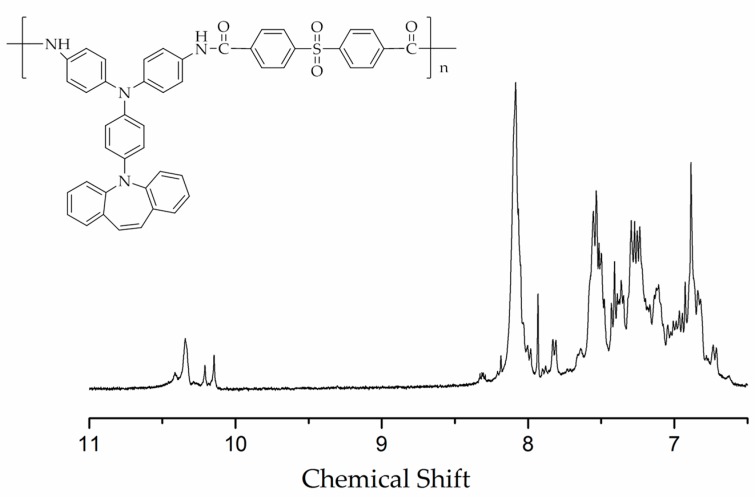
^1^H NMR spectrum of PA–c in DMSO-*d*_6_.

**Figure A7:**
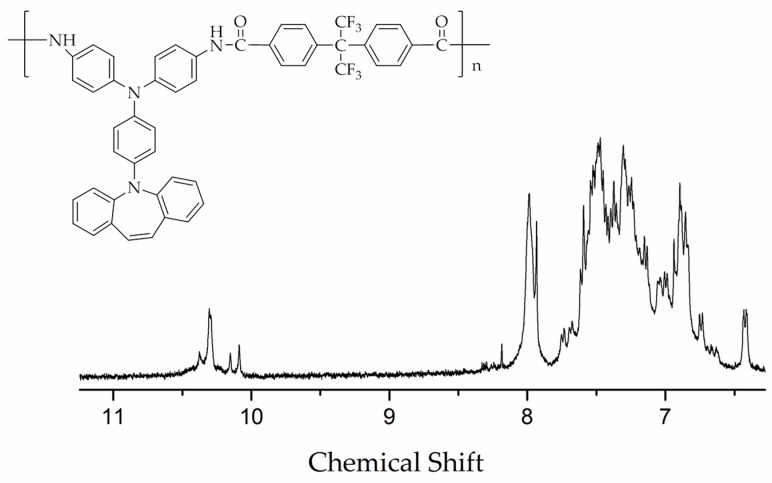
^1^H NMR spectrum of PA–d in DMSO-*d*_6_.

**Figure A8:**
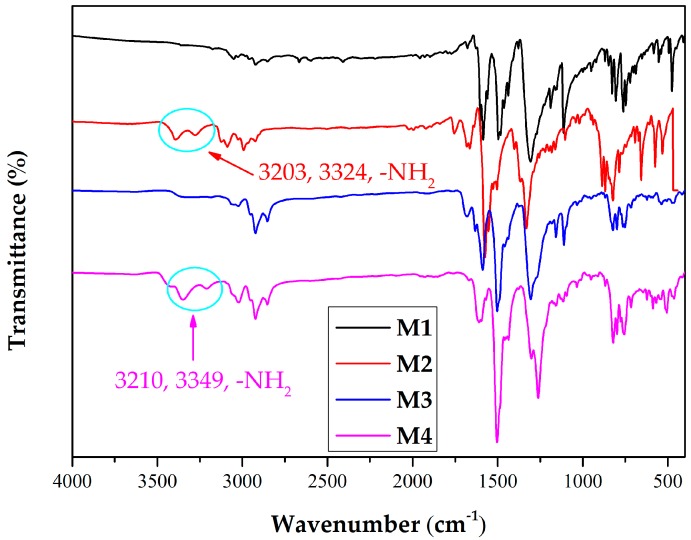
IR of monomers.

**Figure A9:**
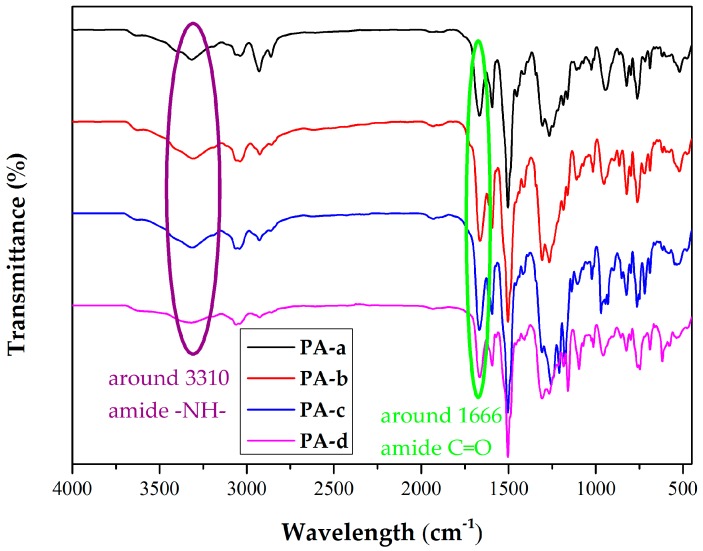
IR of PAs.

According to the phosphorylation technique first described by Yamazaki and co-workers (as shown in Scheme 2), we synthesized a series of novel PAs by using the same diamine monomer M4 with dicarboxylic acids a–d with triphenyl phosphite and pyridine as the condensing agents. After the raw materials were put into the reactor, the solution became viscous. We poured the solution into methanol, then the PAs precipitated in a fiber-like form. IR and NMR spectroscopy have confirmed that PAs have been successfully synthesized. The synthesis of PA–a, used as an example, exhibited the characteristic IR absorption bands of the amide group at 1667 (amide C=O) and 3315 (amide N–H) cm^−1^ (Figure A9). The PAs were also confirmed by ^1^H NMR spectra, with amide resonance peaks appearing around 9.0–11.0 ppm (Figure A4, Figure A5, Figure A6 and Figure A7).

### 3.2. Solubility and Thermal Properties

The solubility tests of these PAs in several organic solvents were investigated by dissolving 20 mg sample in 2 mL of organic solvents. These PAs exhibited excellent solubility in polar organic solvents. All the PAs could be dissolved in polar solvents such as NMP, *N*,*N*-dimethylacetamide (DMAc), DMF, and dimethyl sulfoxide (DMSO) at room temperature, which is favorable for fabricating large-area thin film devices through convenient spin-coating processes for practical applications.

**Figure A10:**
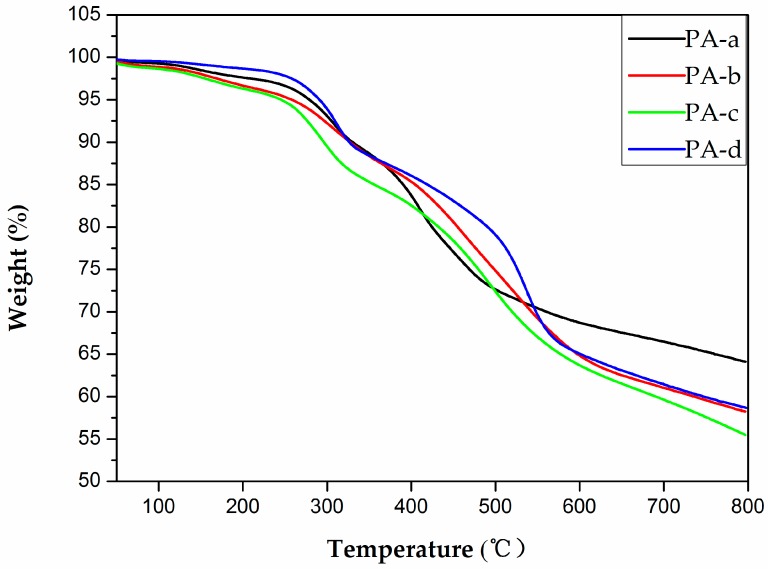
TGA of PAs.

The thermal properties of these PAs were investigated by TGA measurements. In addition, the thermal performance data of the polymer are shown in Table 1. During the decomposition processes in the TGA curves (Figure A10), the 10% weight losses temperatures in nitrogen atmospheres were recorded in the range of 294–326 °C. The char yield was 55–64%. Such high char yield is due to the numerous aromatic rings in the polymeric construction. For four different PAs, the order of *T*_d_ and char yield is presented: PA–a = PA–b = PA–d > PA–c and PA–a > PA–d > PA–b > PA–c. So the stability of the polymer PA–c is worse than the other three. The reason for this phenomenon may be that the rigidity of PA backbone is weakened due to the existence of –CF_3_. Although the C–F bond of the PA–c is strong, the –CF_3_ group falls off when heated. *M*_w_, *M*_n_ and polydispersity (PDI) of PAs were determined by GPC technology.

### 3.3. Optical Properties and Electrochromic Properties

**Figure 2:**
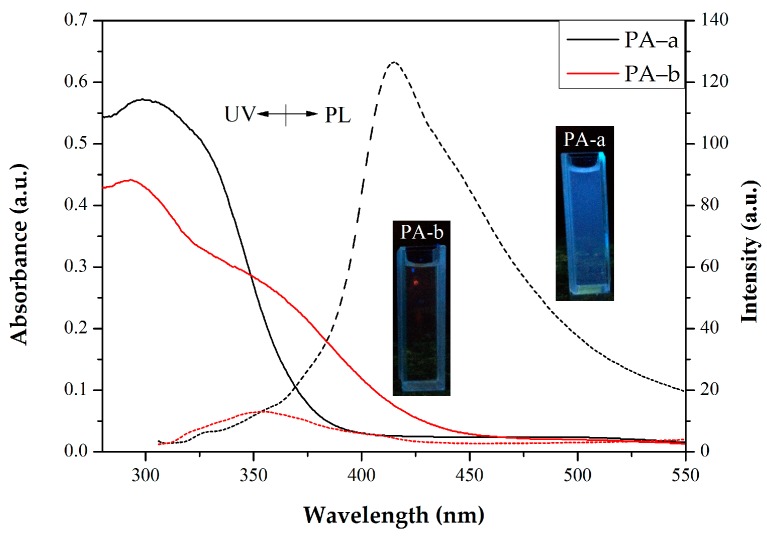
UV-vis (the solid line) and PL (the dotted line) spectra of the PA–a and PA–b in DMSO solutions (1 × 10^−5^ M) with inset taken under irradiation of 365 nm UV light.

In this part, the optical properties of PAs are investigated by UV-Vis and photoluminescence (PL) techniques and the PA–a and PA–b are taken as two representative PAs. As shown in Table 2, the maximum absorption bands of the PAs in solution (DMSO, 1 × 10^−5^ M) and the PAs in films were located at 291–299 nm and 304–352 nm, respectively, which is caused by the π–π * transition of the TPA structure [36,37,38]. In Figure 2, the PA–a solution emitted a stronger fluorescence than the PA–b solution at 365 nm UV lamp irradiation and the semi-aromatic polymer PA–a (Φ_PL_ = 14.94%) exhibited a higher fluorescence quantum yield as compared to the aromatic polymer PA–b (Φ_PL_ = 0.93%). The cause for this phenomenon is that the aliphatic structure on the PA–a backbone effectively reduces the charge transfer.

**Figure 3:**
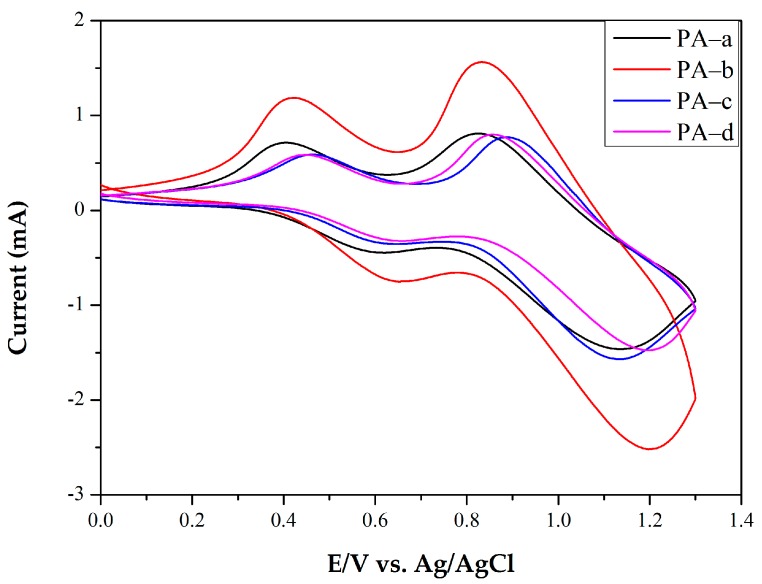
CV diagrams of PAs coated on ITO-coated glass slides in 0.1 M Bu_4_NClO_4_/ACN solution at a scan rate of 50 mV s^−1^.

The results of CV testing are summarized in Table 3. As shown in Figure 3, two pairs of reversible redox waves were observed in these polymers. PA–a is used as an example to illustrate electrochemical performance. When the voltage reached 0.62 V, the first oxidation peak observed can be ascribed to oxidation of the electron-rich nitrogen atom in the TPA core and the film changing from light yellow to green. When the voltage reached 1.13 V, a second oxidation peak was observed. Upon oxidation, the polymer film changed color from green to dark blue. Furthermore, these polymers have onset potentials of the oxidation process range from 0.37 to 0.42 V. The *E*_onset_ value is lower for PAs than other double TPA structures [38].

### 3.4. Quantum Chemistry Calculation

**Figure 4:**
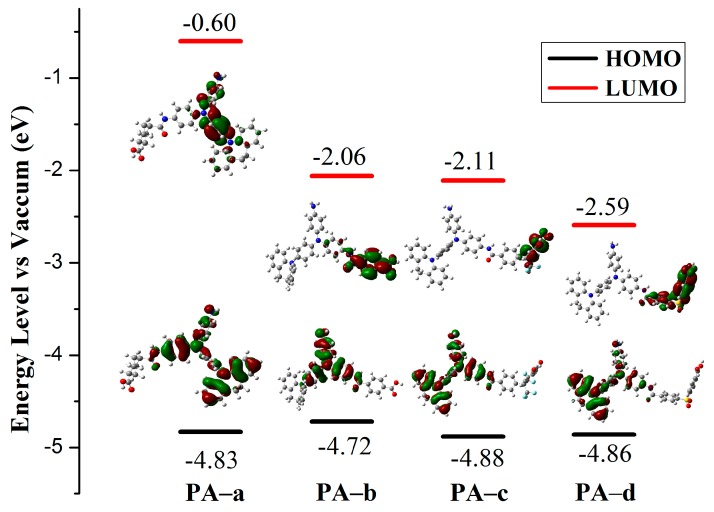
The frontier molecular orbitals computed for PAs at the B3LYP/6-31G.

The HOMO and LUMO values for these PAs were calculated to be in the range of −4.87 to −4.91 eV and −1.72 to −2.24 eV, respectively. However, as shown in Figure 4, the theoretical calculation was calculated to be the range of −4.72~−4.88 eV and −0.60~−2.59 eV. This bias may be due to the fact that the theoretical data is calculated for the monomer, not the long chain polymer. In addition, the experimental data were obtained from polymers, which are influenced by the interaction of solvent and electrolyte.

### 3.5. Spectroelectrochemical and Electrochromic Properties

The electrochromism properties of PA films were investigated by UV-Vis–NIR (Near Infrared) spectroscopy, and their absorption profiles were monitored with a UV-Vis spectrometer at different applied potentials. The electrode preparations and solution conditions were identical to those used in CV. The films of all PAs exhibited strong absorption at wavelengths around 304 to 412 nm in the neutral form. Upon oxidation, the main band located at around 390 to 918 nm grew.

**Figure 5:**
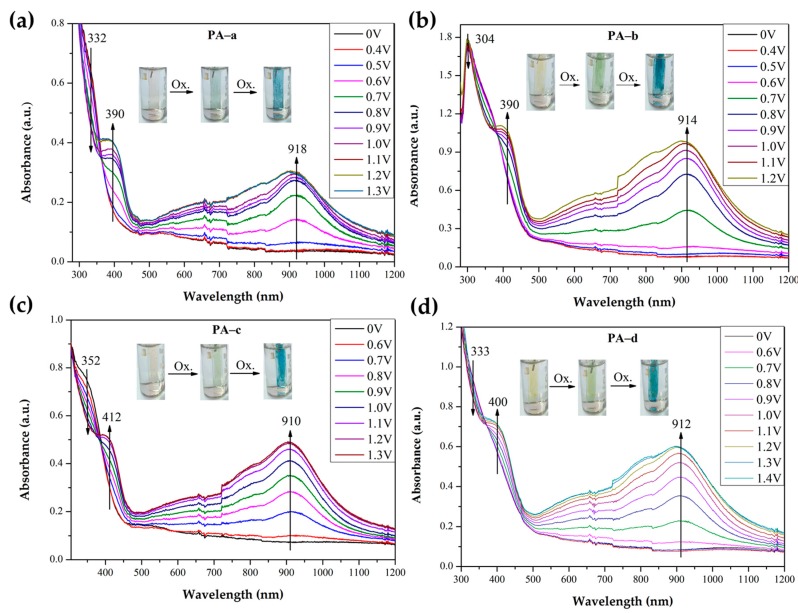
The electronic absorption spectrum of film of PA–a (**a**); PA–b (**b**); PA–c (**c**) and PA–d (**d**) was carried out in 0.1 M Bu_4_NClO_4_/ACN as the supporting electrolyte.

As a typical example, the spectral changes of PA–b at various applied potentials are shown in Figure 5. In the neutral form, the film exhibited strong absorption at wavelengths around 304 nm due to the π–π * transition of the TPA group. When the applied potentials increased from 0 to 0.6 V, the absorption peak of PA at 304 nm decreased gradually, whereastwo new bands grew at 390 and 914 nm, and the absorption at 914 nm was caused by the charge transfer effect (IVCT). As the applied voltage was raised to 1.2 V, the resulting spectrum did not change significantly due to TPA having been completely oxidized.

**Figure 6:**
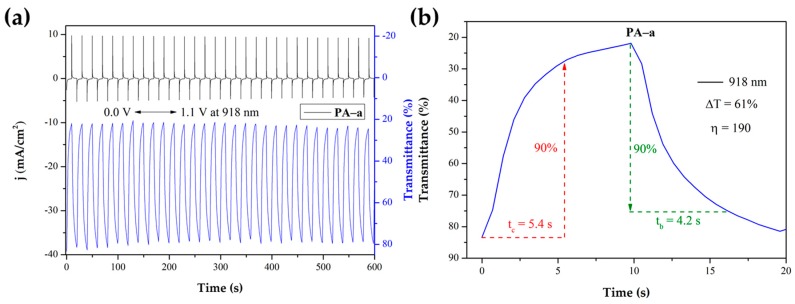
Dynamic changes of the transmittance and current (**a**) and optical switching (**b**) of PA–a.

The stability, response time, and color efficiency are important parameters for an electroactive polymer film, thus it is necessary for the electrochromic switching to be studied further. We carried out electrochromic switching studies for the PA films to record the percent transmittance changes (ΔT%) of dependence on time at their absorption maximum. The response time is determined by changing the step potential between the neutral and oxidized states, which is required to reach 90% of all the changes in absorbance after switching potential. The relevant time is displayed in Table 4. Figure 6 depict the optical transmittance of polymer films at 918 nm, 914 nm, and 910 nm as a function of time by applying square-wave potential steps between 0 and 1.1 V for a pulse time of 10 s. The electrochromic coloration efficiency (CE: η) is one of the important characteristics of electrochromic materials. CE can be calculated using the related equations. After continuous cyclic scans between 0.0 V and 1.1 V in 600 s, all polymer films still exhibited excellent stability and good stability of electrochromic characteristics, indicating that the film was very stable.

Take a typical example, the PA–a revealed a satisfactory switching time of 5.4/4.2 s for the coloring/blenching process. The optical contrast, measured as Δ*T*% between neutral and oxidized state, was found to be 61% for PA–a. The CE of PA–a film was calculated to be 190 cm^2^ /C (at 918 nm).

### 3.6. Photoelectrical Properties

**Figure 7:**
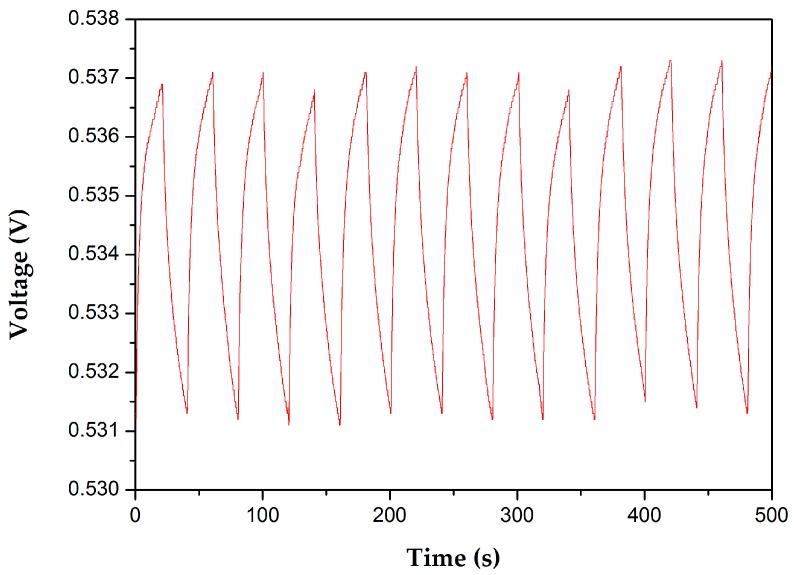
A typical photovoltaic response for PA–c film immobilized on ITO glass upon exposure to light with switching at room temperature.

**Figure A11:**
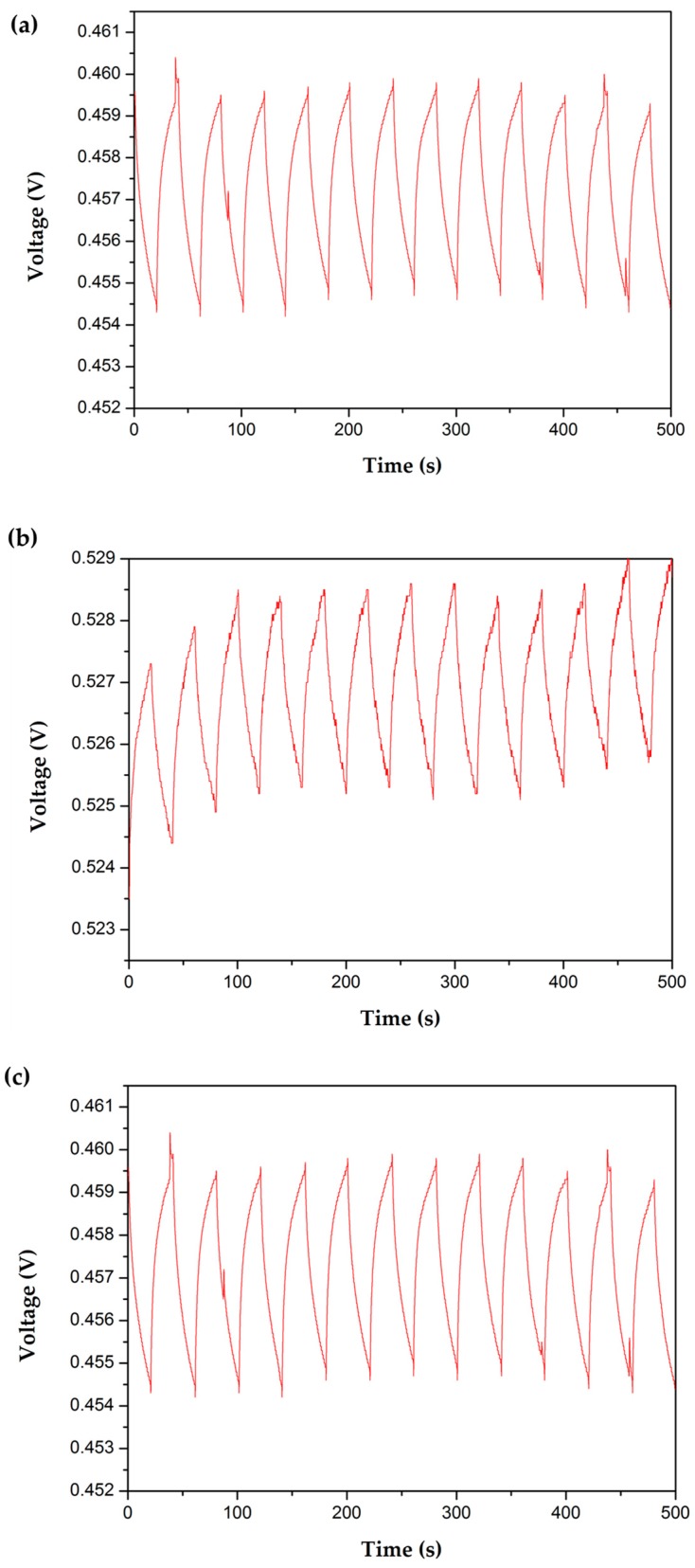
A typical photovoltaic response for PA–a (**a**); PA–b (**b**); and PA–d (**c**) film immobilized on ITO glass upon exposure to light with switching at room temperature.

To enlarge the application range of PAs, we further studied the photovoltage characteristics of the PAs. Switching the light on and off many times determined the reversible rise/decay process of the photovoltage response. We placed the films in a 0.2 M Bu4NClO_4_/ACN electrolyte solution and illuminated them with a 500 W xenon arc lamp (white light intensity of 150 mW cm^−2^). As shown in Figure 7, the photoelectric response was stable despite the light switching on and off many times. When the light is turned on, the photoelectric voltage immediately increases to the maximum. On the contrary, after the light is turned off, the photoelectric voltage drops sharply to the original state. It can be observed that other PAs have a similar trend (see Figure A11). The observation result can be explained by the fact that the photo-generated electrons are transported from the lowest unoccupied molecular orbital to the conduction band of the ITO surface, and then moved to the external circuit, where the electron migration causes the change in photovoltage [39]. Therefore, these PAs can be potential materials in the photoelectric conversion of material or photodetector fields.

### 3.7. Electrofluorochromic Performance

**Figure 8:**
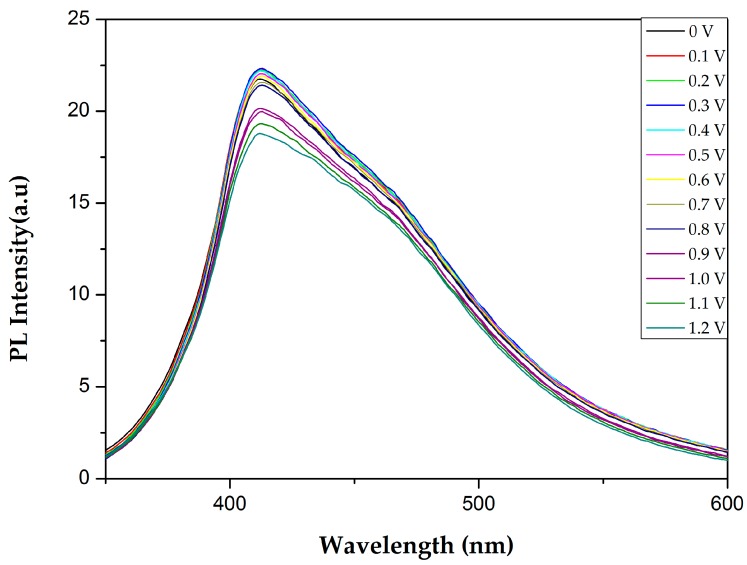
Fluorescence spectra changes of PA–a film upon different applied potentials from 0.0 to 1.2 V.

**Figure A12:**
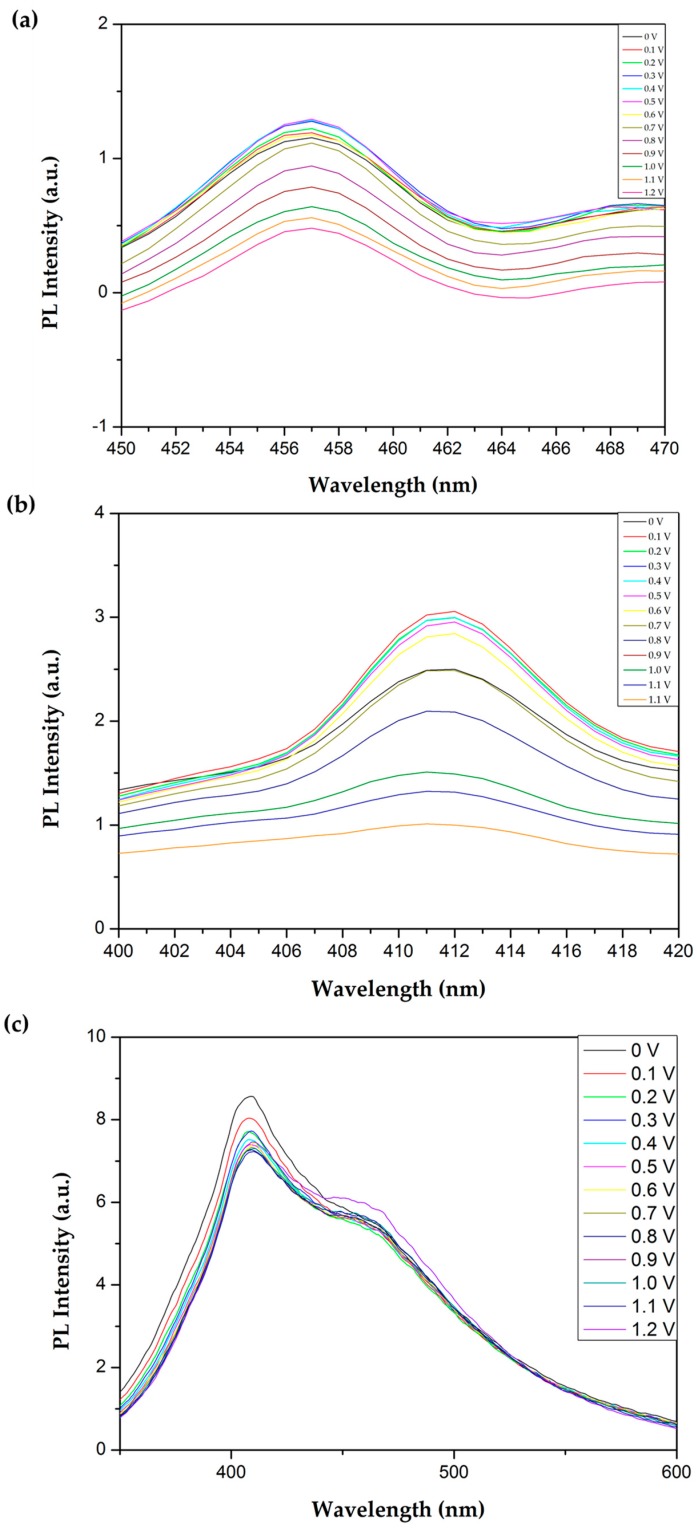
Fluorescence spectra changes of PA–b (**a**); PA–c (**b**); and PA–d (**c**) film upon different applied potentials from 0.0 to 1.2 V.

Electrofluorescent measurements were carried out to evaluate the optical properties in the way that Sun has reported [25]. As shown in Figure 8, the fluorescence changes under a series of positive potentials recorded to demonstrate its electrofluorochromic switching properties. The dynamic response behavior was examined by oxidation steps between 0.0 and 1.2 V. When we gradually increased the voltage, the fluorescence value of the polymer changed. A similar trend was observed for the other PAs (Figure A12). This can be attributed to the changes of the effective fluorescence quencher (TPA^+^) in the polymer.

## 4. Conclusions

In summary, a series of novel electroactive PAs with 5*H*-dibenzo[*b,f*]azepin-5-yl-substituted triphenylamine have been successfully synthesized. They all exhibited good solubility in common organic solvents as well as excellent electrochemical properties, and could afford flexible and strong films with good mechanical properties. The introduction of a 5*H*-dibenzo[*b,f*]azepin-5-yl group substituent on the TPA unit greatly lowered the oxidation potentials of the PAs and enhanced the electrochemical and electrochromic stability of the PAs. From the CV curve can be seen two obvious pairs of redox peaks, and the process of oxidation from yellow to green and then into a dark blue. These polymers also revealed high optical contrast, high coloration efficiency, and good cycling stability. Thus, these new PAs can be good candidates for use in optoelectronics applications.

## Figures and Tables

**Table 1:** Thermal properties and molecular weights of the PAs.

Polymer Code	*T*_d_ (°C) 10%	Char Yield (%)	*M*_w_ (Da)	*M*_n_ (Da)	PDI
PA–a	326	64	1.40 × 10^4^	1.11 × 10^4^	1.26
PA–b	326	58	1.33 × 10^4^	1.05 × 10^4^	1.27
PA–c	294	55	1.58 × 10^4^	1.34 × 10^4^	1.18
PA–d	326	59	1.34 × 10^4^	1.12 × 10^4^	1.20

**Table 2:** Optical properties of PAs.

Polymer Code	In Solution λ (nm)			As Film λ (nm)	
	Abs Max	PL Max	ФPL (%)	Abs Max	Abs Onset
PA–a	299	415	14.94	332	394
PA–b	293	354	0.93	304	468
PA–c	291	352	1.12	352	436
PA–d	293	352	0.96	333	430

**Table 3:** Electrochemical properties of PAs.

Polymer Code	Oxidation Potential (V)			*E*_g_^opt^ (eV) ^c^	Energy Levels (eV)	
	*E*_onset_	*E*_1/2_^ox1^	*E*_1/2_^ox2^		HOMO ^a^	LUMO ^b^
PA–a	0.37	0.52	0.98	3.15	−4.87	−1.72
PA–b	0.39	0.54	1.02	2.65	−4.89	−2.24
PA–c	0.42	0.56	1.02	2.84	−4.91	−2.07
PA–d	0.42	0.56	1.02	2.88	−4.91	−2.03

^a^ *E*_HOMO_ = −(*E*_1/2_ vs. Ag/AgCl + 4.80 − *E*_1/2,ferrocene_) eV. Ferrocene was used as an external reference for calibration (*E*_1/2,ferrocene_ = +0.45 V vs. Ag/AgCl) in Bu_4_NClO_4_/ACN; ^b^ *E*_LUMO_ = *E*_HOMO_ + *E*_g_^opt^; ^c^ *E*_g_^opt^ = 1240/λ_onset_.

**Table 4:** Electrochromic properties of PAs.

Polymer Code	λ_max_ (nm)	ΔT (%)	Response Time		ΔOD	Q_d_ (mC cm^−2^)	CE (cm^2^ C^−1^)
			t_c_ (s)	t_b_ (s)			
PA–a	918	61	5.4	4.2	0.580	3.059	190
PA–b	914	74	5.7	4.7	0.820	3.987	206
PA–c	910	58	4.9	5.8	0.599	2.636	227
PA–d	912	70	6.1	5.5	0.822	3.172	259
